# Tetramethylpyrazine: An Active Ingredient of Chinese Herbal Medicine With Therapeutic Potential in Acute Kidney Injury and Renal Fibrosis

**DOI:** 10.3389/fphar.2022.820071

**Published:** 2022-01-25

**Authors:** Jun Li, Xuezhong Gong

**Affiliations:** Department of Nephrology, Shanghai Municipal Hospital of Traditional Chinese Medicine, Shanghai University of Traditional Chinese Medicine, Shanghai, China

**Keywords:** tetramethylpyrazine, acute kidney injury, renal fibrosis, Chinese medicine, mechanism

## Abstract

As an increasing public health concern worldwide, acute kidney injury (AKI) is characterized by rapid deterioration of kidney function. Although continuous renal replacement therapy (CRRT) could be used to treat severe AKI, effective drug treatment methods for AKI are largely lacking. Tetramethylpyrazine (TMP) is an active ingredient of Chinese herb *Ligusticum wallichii* (*Chuan Xiong*) with antioxidant and anti-inflammatory functions. In recent years, more and more clinical and experimental studies suggest that TMP might effectively prevent AKI. The present article reviews the potential mechanisms of TMP against AKI. Through search and review, a total of 23 studies were finally included. Our results indicate that the undergoing mechanisms of TMP preventing AKI are mainly related to reducing oxidative stress injury, inhibiting inflammation, preventing apoptosis of intrinsic renal cells, and regulating autophagy. Meanwhile, given that AKI and chronic kidney disease (CKD) are very tightly linked by each other, and AKI is also an important inducement of CKD, we thus summarized the potential of TMP impeding the progression of CKD through anti-renal fibrosis.

## Introduction

Acute kidney injury (AKI) is characterized by an abrupt loss of renal function, mainly manifested by increased serum creatinine (sCr) levels and decreased urine output. The duration of AKI is generally less than 7 days, and the functional criteria are: increase in sCr by ≥50% within 7 days or increase in sCr by ≥ 0.3 mg/dl (26.5 μmol/L) within 2 days or oliguria for ≥6 h (Khwaja, 2012). A meta-analysis combined research data from 3,585,911 people from most areas north of the equator. The results reported that the combined morbidity and related mortality of AKI in adults were 21.6% and 23.9%, respectively, and 33.7% and 13.8% in children, respectively (Susantitaphong et al., 2013). Due to different medical resources, the cause and incidence of AKI vary greatly among different countries. In high-income countries, AKI is mostly hospital-acquired, mainly in elderly patients with multiple organ failure. In low- and middle-income countries, AKI mainly occurs as a complication of a single disease, and approximately 77% of AKI is community-acquired (Mehta et al., 2015; Hoste et al., 2018). The global burden of AKI-related mortality has exceeded the burden of breast cancer, heart failure or diabetes, and its medical burden is increasing (Lewington et al., 2013). In addition, AKI is associated with progressive chronic kidney disease (CKD) and the following end-stage renal disease (ESRD), which further aggravates the harm of AKI. Many studies have reported some chemical and biological agents have beneficial effects on AKI but there is still a lack of accepted therapeutic drugs so far (Yang et al., 2016; Ronco et al., 2019). AKI patients not only have an increased risk of recent mortality and cardiovascular events, but also have a long-term risk of CKD (See et al., 2019). After the occurrence of AKI, if the kidney tissue is repaired excessively, repaired incompletely, or the damage persists, it might lead to renal dysfunction and renal fibrosis. The progression of AKI to CKD is a complex process involving the regulation of multiple cells and multiple signaling pathways, such as inflammatory damage, G2/M cell cycle arrest, oxidative stress and apoptosis, and these processes ultimately lead to or aggravate renal fibrosis (Vernon et al., 2010; He et al., 2017; Liu et al., 2017; Dong et al., 2018).

**FIGURE 1:**
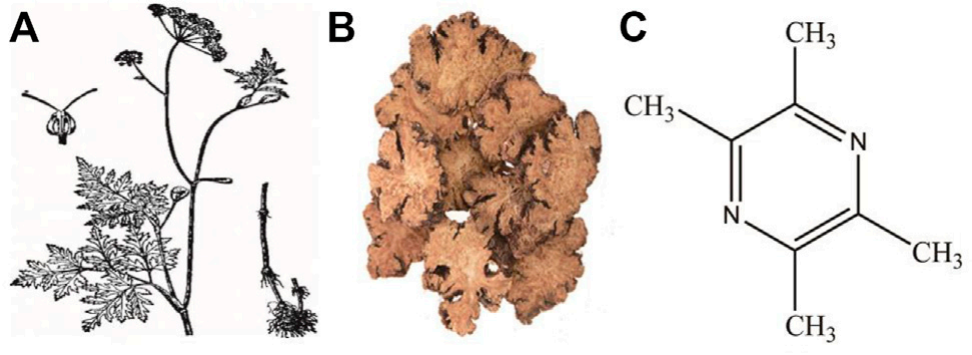
Illustration of Ligusticum wallichii plant **(A)**, decoction pieces **(B)** and chemical structure of TMP **(C)**.

Tetramethylpyrazine (ligustrazine, TMP) is the active ingredient and characteristic alkaloid of the Chinese herbal medicine *Ligusticum wallichii* (*Chuan Xiong*) (Figure 1). TMP has the effects of inhibiting platelet aggregation, reducing blood viscosity, increasing coronary flow, scavenging free radicals, protecting cerebral vessels, and expanding renal vessels (Zou et al., 2018). The pyrazine ring on the TMP molecule is the key group for its pharmacological effect, but the methyl group in its side chain is easily excreted by oxidative metabolism, which leads to the short half-life of TMP and weakens its pharmacological effect (Wang et al., 2019). Pharmacokinetic studies have shown that after oral or intravenous injection, TMP is mainly distributed in tissues such as liver, brain, kidney, and small intestine, and is eventually excreted from urine through the kidney (Lou et al., 1986; Pan et al., 2021). In view of its anti-oxidative and anti-inflammatory effects, TMP is widely used in cardiovascular and cerebrovascular diseases (Zhao et al., 2016). To date, many studies have focused on the benefits of TMP in a variety of animal or cell models of AKI (Li et al., 2019). Through years of exploration, our team has also confirmed that TMP and Chinese herbal formulas containing *Chuan Xiong* have an intervention effect in AKI caused by contrast mediums (Gong, 2018; Norgren and Gong, 2018; Gong, 2020). TMP also could function as anti-renal fibrosis and be used in clinical to treat renal fibrosis and CKD. Although there are many reports on TMP effects in AKI, no systematic summary is available. Based on the evaluation of the evidence supporting this hypothesis, we mainly reviewed the therapeutic effects and mechanisms of TMP on AKI. Considering the close relationship between AKI and CKD, the present study also briefly summarized the effects of TMP on renal fibrosis and CKD.

## Categories and Pathology of AKI

**FIGURE 2:**
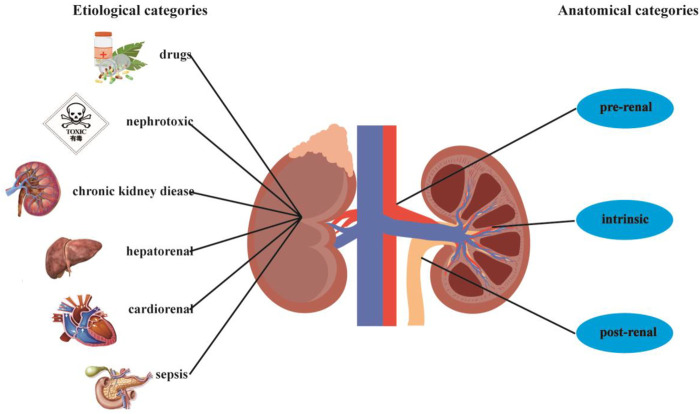
Categories of the causes of AKI.

Although the cause of the disease is extremely complex, AKI is usually regarded as a single disease. Generally, it is divided into three categories based on anatomical location: pre-renal, intrinsic, and post-renal. In recent years, this simple classification method of AKI has been replaced by more specific etiological categories, since different etiologies often mean different pathological mechanisms (Bellomo et al., 2012). Related causes in the latter etiological categories include drugs, sepsis, toxins, cardiorenal, obstruction, hepatorenal, and renal hypoperfusion (Figure 2) (Privratsky et al., 2018; Huang et al., 2019; Jentzer et al., 2020; Simonetto et al., 2020; Molema et al., 2021). In terms of pathological manifestations, AKI is generally described as damage to renal tubular epithelial cells and vascular system (Linkermann et al., 2014; Sancho-Martínez et al., 2015). Due to pathological factors, a variety of stresses occur in AKI, including hypoxia, nutrient deprivation, energy consumption, oxidative damage, genotoxic stress, and endoplasmic reticulum stress. These stresses eventually affect renal tubular epithelial cells by causing oxidative stress damage, inflammation, necrosis, mitochondrial dysfunction, apoptosis, and autophagy (Sureshbabu et al., 2015; Cybulsky 2017; Kimura et al., 2017). Renal hypoperfusion is due to the lack of oxygen and nutrition in the nephrons, which activates the damage and death of epithelial cells through necrosis or apoptosis, ultimately leading to endothelial injury, inflammatory activation, and renal dysfunction (Makris and Spanou 2016). Nephrotoxic drugs and toxins have direct cytotoxic effects on renal tubular epithelial cells and endothelial cells. In addition, they impair hemodynamics and deposit metabolites (Yatim and Oberbarnscheidt, 2015; Wu and Huang, 2018). In sepsis, the reduction of effective circulating blood volume leads to a reduction in renal blood flow and oxygen delivery. Simultaneously, it is accompanied by immune inflammation and activation of the coagulation cascade (Peerapornratana et al., 2019). Although the mechanisms of renal hypoperfusion, nephrotoxic drugs, sepsis, and other causes of AKI are different, they all involve the pathophysiological links of hemodynamic changes, oxidative stress injury, and inflammation.

## Methods

**FIGURE 3:**
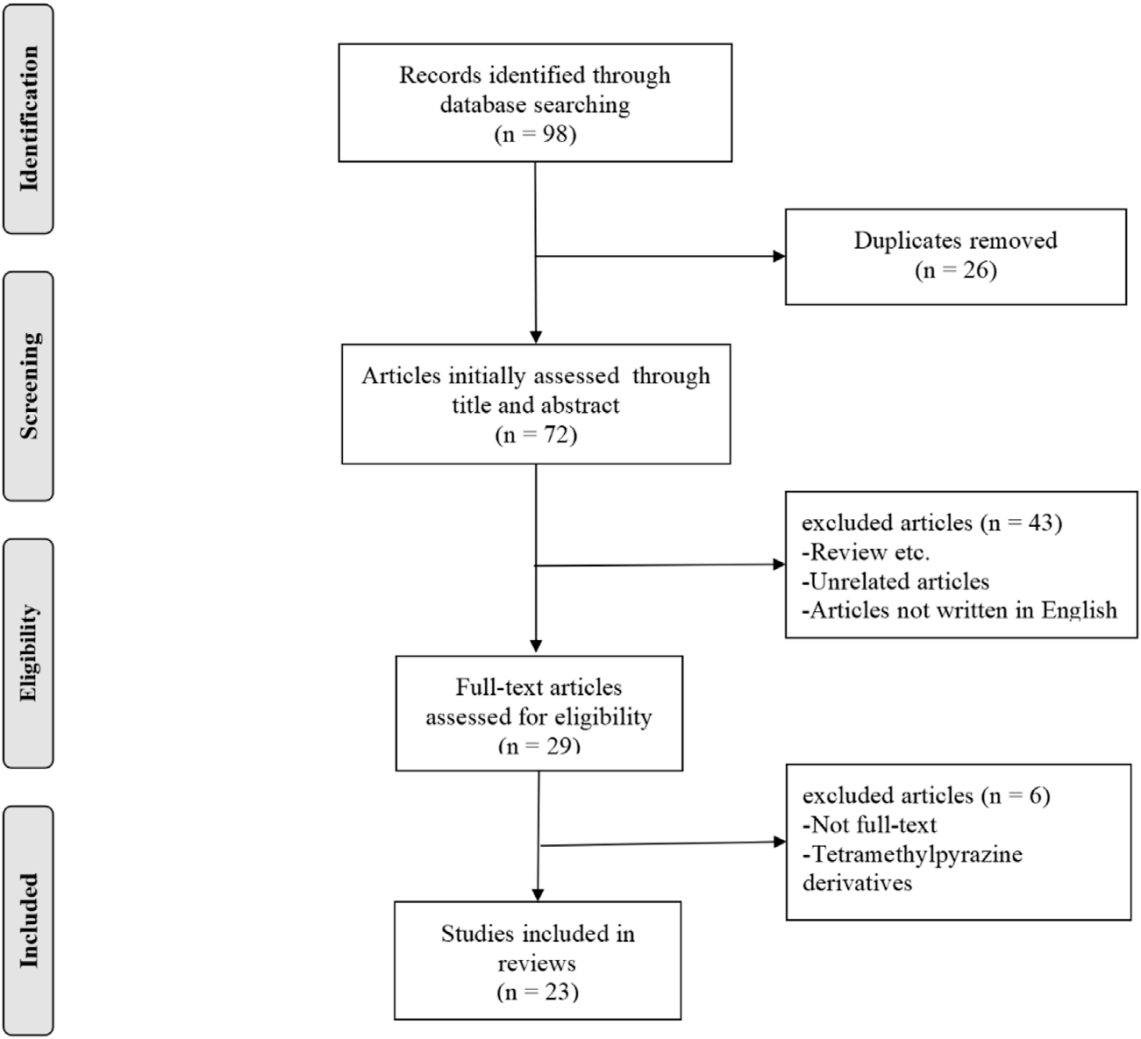
Summary of the literature search process.

This study required a systematic search of electronic databases to identify studies to determine the renal protective effect of TMP on AKI. The search was performed using PubMed and Embase. The following combination of terms were used as search keywords: “Tetramethylpyrazine” OR “Ligustrazine” AND “kidney injury” OR “Renal Injury” OR “Nephrotoxicity” OR “Renal ischemia.” The specified exclusion criteria included: a) case reports, clinical studies, case series, editorials, and reviews; b) research on tetramethylpyrazine derivatives; and c) articles not written in English. A summary of the literature search process is presented in Figure 3.

## Results

### Studies Characteristics and Mechanism

**FIGURE 4:**
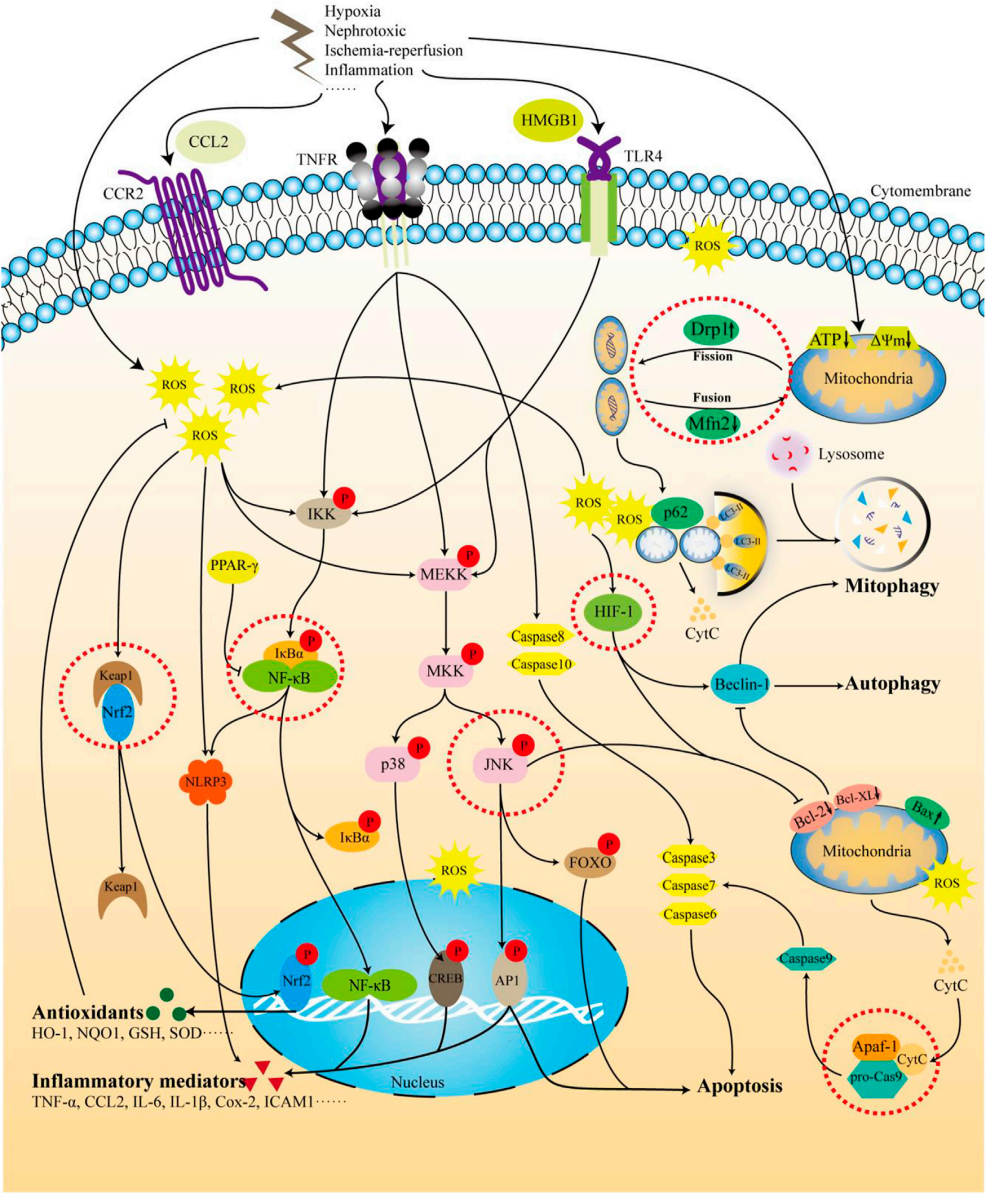
The mechanism of TMP intervention in AKI. The figure summarizes the molecular pathways of TMP treatment of AKI involved in this review. Receptors such as TNR, TLR, and CCR2 are stimulated by nephrotoxic drugs, LPS, I/R, and inflammatory factors. In addition, hypoxia and I/R can also directly affect the mitochondrial quality control process and membrane potential, leading to the generation of ROS. The activation of the above receptors and the production of intracellular ROS can activate downstream pathways, further triggering inflammation, apoptosis, and autophagy, and ultimately leading to kidney damage. TMP can target Nfr2 and HIF-1 to activate the expression of antioxidant factors and enhance cell tolerance to oxidative stress. TMP can also inhibit TLR4 and TNFR or, by activating PPAR-γ, further inhibit the NF-κB pathway and reduce inflammation. In addition to the targeted inhibition of caspase-8/3/6/7 through the TNFR pathway, TMP can also affect mitochondrial-related apoptosis by inhibiting the ERK/JNK pathway. There is still controversy regarding the regulation of autophagy by TMP. It is generally believed that TMP activates the autophagy process and eliminates damaged mitochondria by targeting mitochondrial quality control, ultimately reducing cell damage.

In total, 98 potentially relevant studies were screened. Ultimately, 23 experimental studies met the inclusion and exclusion criteria (Table 1). In Table 1, there are 17 *in vivo* studies, 2 *in vitro* studies, and four both. Of the 23 studies, 12 were on nephrotoxic drugs and toxins, seven on ischemia-reperfusion, two on contrast mediums, one on sepsis, and one on severe burns. These studies involve the use of TMP, including oral, intravenous, and intraperitoneal injections. Based on the results of these studies, TMP had a therapeutic effect on AKI caused by a variety of etiologies. In terms of mechanism, TMP could alleviate AKI by reducing oxidative stress, inflammation, mitochondrial and other organelle damage, or affecting cytoprotective mechanisms such as autophagy or apoptosis (Figure 4). The target highlighted by the red dashed line in the figure represents the key link for TMP to exert its effect. These mechanisms are described in detail below.

**TABLE 1:** *In vivo* and *in vitro* studies of TMP intervention AKI.

Type	Animal/Cell	Model	Inducer	TMP	Histological score	Markers	References
*In vivo*	ICR mice	ethanol-induced AKI	absolute ethanol	10, 25, 50 mg/kg; p.o.	No scoring	SrCr↓, BUN↓, MDA↓, Cytc↓	Liu et al. (2002)
*In vivo*	Wistar rats	I/R injury	renal artery clipping + reperfusion	4 ml/kg; i.v.	proximal convoluted tubule: 0 = normal; 1 = mitoses and necrosis of individual cells; 2 = necrosis of all cells in adjacent tubules; 3 = necrosis confined to the distal third of, necrosis across the inner cortex; 4 = necrosis affecting all three segments of tubule	MDA↓, SOD↑, ET-1↓	Sun et al. (2002)
*In vivo*	Wistar rats	I/R injury	hepatic/renal I/R	not clear; i.v.	No scoring	SrCr↓, BUN↓, P-selectin↓	Chen et al. (2003)
*In vivo*	C57BL/6 mice	I/R injury	right nephrectomy + left renal ischemia	80 mg/kg; i.p.	number of necrotic and apoptotic cells, loss of tubular brush border, tubular dilatation, cast formation, and neutrophil infiltration: 0 = none; 1=< 10%; 2 = 11–25%; 3 = 26–45%; 4 = 46–75%; 5=> 76%	SrCr↓, BUN↓, MDA↓, SOD↑, Bcl-2↑, ICAM-1↓	Feng et al. (2004)
*In vivo*	SD rats	ANP-AKI	sodium taurocholate	6 g/L; i.v.	tubular epithelial cells: 0 = normal; 1 = notable cloudy swelling; 2 = swelling denaturation, interstitial congestion, edema and infiltration of inflammatory cells; 3 = diffuse coagulation necrosis	SrCr↓, BUN↓, TXA2/PGI2↓	Zhang et al. (2006)
*In vitro* and *In vivo*	SD rats/NRK-52E cells	DI-AKI	gentamicin	80 mg/kg/d; i.p.	No scoring	Bcl-xL↑, TNF-α↓, NF-κB↓, caspase-3↓, caspase-8↓, caspase-9↓	Juan et al. (2007)
*In vivo*	Wistar rats	DI-AKI	cisplatin	80 mg/kg/d; p.o.	approximate extent of necrotic area in the cortical proximal tubules: 0 = no necrosis; 1 = a few focal necrotic spots; 2 = necrotic area about onehalf; 3 = necrotic spots about two-thirds; 4 = nearly all of the area necrotic	SrCr↓, BUN↓, GSH↑, NAG↓, SOD↑, TOX↑	Ali et al. (2008)
*In vivo*	SD rats	DI-AKI	Cisplatin	50, 100 mg/kg; i.p.	No scoring	SrCr↓, BUN↓, MDA↓, NAG↓, SOD↑, GSH↑, GST↑, NOS↓, NO↓	Liu et al. (2008)
*In vivo*	Wistar rats	DI-AKI	Gentamicin	100 mg/kg/d; p.o.	No scoring	SrCr↓, BUN↓, UNAG↓	Ali et al. (2009)
*In vivo* and *In vitro*	C57B6 mice/NRK-52E	DI-AKI	gentamicin	80 mg/kg/d; i.p.	tubular necrosis: 0 = normal; 1 ≤ 10%; 2 = 10–25%; 3 = 26–75%; 4 ≥ 75% cells exhibiting necrosis	HO-1↑, Bcl-xL↑, Hax-1↑, NADPH↓, NF-κB↓, Cox-2↓, caspases-3↓, caspases-9↓	Sue et al. (2009)
*In vivo*	C57BL/6 mice	I/R injury	renal artery clipping + reperfusion	80 mg/kg; i.p.	positive tubular brush border, tubular dilatation, cast formation, neutrophil infiltration: 0 = none; 1 = 10%; 2 = 11–25%; 3 = 26–45%; 4 = 46–75%; 5 = 76%	MPO↓, MDA↓, SOD↑, TNF-α↓, ICAM-1↓	Feng et al. (2011)
*In vivo*	Lewis rats	severe burn	30% TBSA scald injury	40 mg/kg/d; i.p.	expression of Bcl-2 and MICA: 0 = 0–5% stained; 1=> 5–25%; 2=> 25–50%; 3=> 50–75%; 4=> 75%	MDA↓, SOD↑, MICA↓, Bcl-2↓	Gao et al. (2012)
*In vivo*	SD rats	CIN	L-NAME + indomethacin + iohexol	80 mg/kg/d; i.p.	No scoring	SrCr↓, BUN↓, phospho-p38 MAPK↓, FoxO1↓, Bcl-2↑, Bax↓, iNOS↓, CysC↓, UNAG↓, UGGT↓	Gong et al. (2013)
*In vivo*	SD rats	DI-AKI	Cadmium chloride (CdCl2)	50 mg/kg; i.p.	No scoring	BUN↓, kim-1↓, indoxyl sulfate↓, clusterin↓, MDA↓, SOD↓, GR↓, LDH↓, ALP↓	Lan et al. (2014)
*In vitro*	HK-2 cells	DI-AKI	sodium arsenite	—	No scoring	ROS↓, GSH↑, β-catenin↓, NF-κB↓, p38 MAPK↓, COX-2↓, TNF-α↓, Cytc oxidase↑, mitochondrial membrane potential↑	Gong et al. (2015)
*In vitro*	HK-2 cells	DI-AKI	sodium arsenite	—	No scoring	HO-1↓, ARS2↓ p38 MAPK↓, JNK↓, AP-1↓, Nrf2↓, NF-κB↓	Gong et al. (2016)
*In vivo*	SD rats	DI-AKI	Cadmium chloride (CdCl2)	50 mg/kg; i.p.	No scoring	SrCr↓, BUN↓, MDA↓, 4-HNE↓, GSH↑, GSH/GSSG↑, SAM↑, cystathionine↑, MATs↑, CBS↑	Kuang et al. (2017)
*In vivo*	SD rats	CIN	L-NAME + indomethacin + iohexol	80 mg/kg/d; i.p.	No scoring	SrCr↓, BUN↓, Drp1↓, Mfn2↑, CCL2↓, CCR2↓, LC3B-II/I↓, Beclin-1↓, p62↑, procaspase 9↑, caspase 3↓, TNF-α↓, ROS↓, IL-6↓, CysC↓, UNAG↓, UGGT↓	Gong et al. (2019)
*In vivo*	SD rats	DI-AKI	Cisplatin	50, 100 mg/kg/d; i.p.	No scoring	SrCr↓, BUN↓, HMGB1↓, TLR4↓, NF-κB↓, TNF-α↓, IL-1β↓, GSH↑, SOD↑, PPAR-γ↑, Nrf2↑, Bax↓, Bcl2↑, caspase-3↓, HO-1↑, NQO1↑, COX-2↓, iNOS↓, Kim-1↓	Michel and Menze, (2019)
*In vivo*	C57BL/6 mice	Sepsis-AKI	cecal ligation and puncture (CLP)	10, 30, 60 mg/kg; i.v.	pathological changes of renal cortex or outer zone of medulla: 0 = normal; 1 = less than 5%; 2 = 5–25%; 3 = 25–75%; 4= > 75%	Kim1↓, caspase- 3↓, NMDAR1↓	Ying et al. (2020)
*In vivo*	SD rats	I/R injury	renal artery clipping + reperfusion	40 mg/kg; i.p.	renal tubular injury: 1 = normal; 2 = 0–10%; 3 = 11–25%; 4 = 26–45%; 5 = 46–75%; 6= > 75%	TNF-α↓, IL-1β↓, IL-6↓, MDA↓, GSH↑, LC3B-II/I↑, Beclin-1↑	Chen et al. (2020)
*In vivo* and *In vitro*	SD rats/NRK-52E cells	I/R injury	renal artery clipping + reperfusion/CoCl2/OGD + reoxygenation	40 mg/kg; i.p.	injury in tubules of the outer medulla: 0 = none; 1 = 0–10%; 2 = 11–25%; 3 = 26–45%; 4 = 46–75%; 5=> 75%	SrCr↓, BUN↓, NOD2↓, TNF-α↓, IL-6↓, MCP-1↓, caspase-3/cleaved caspase-3↓, LC3A/B-II/I↑	Jiang et al. (2020)
*In vivo* and *In vitro*	SD rats/NRK-52E cells	I/R injury	renal artery clipping + reperfusion/OGD + reoxygenation	200 mg/kg; p.o.	No scoring	SrCr↓, BUN↓, TNF-α↓, IL-6↓, NLRP3↓, HIF-1α↓, KIM-1↓	Sun et al. (2020)

### TMP Relieves Oxidative Stress Injury

Oxidative stress reflects a state of imbalance between the formation of reactive oxygen and nitrogen and antioxidant system. Oxidative stress occurs when the production of pro-oxidants or reactive oxygen species (ROS) exceeds the endogenous antioxidant capacity (Sies et al., 2017). ROS are several active molecules and free radicals derived from molecular oxygen, including superoxide anions (O2^−^) and hydroxyl radicals (OH). At high concentrations, ROS can be toxic to macromolecules, including lipids, proteins, and DNA, leading to the destruction of the integrity and capacity of the cell structure (Davies, 1987; Sies, 1997). Oxidative stress is an important pathological mechanism of AKI caused by various etiologies. In AKI induced by ischemia-reperfusion injury, sepsis, and contrast mediums, changes in renal hemodynamics can lead to increased ROS production. In the hypoxic state, electron transfer in the mitochondrial respiratory chain is obstructed, causing electron leakage. The leaked electrons combine with oxygen to generate a large amount of active oxygen (Granata et al., 2015; Kusirisin et al., 2020). Cisplatin and aminoglycoside drugs can induce mitochondrial dysfunction and increase the production of ROS and can also react with thiol-containing molecules, including glutathione (GSH). The consumption or inactivation of GSH and related antioxidants leads to the intracellular accumulation of endogenous ROS (Kruidering et al., 1997; Sureshbabu et al., 2015). Other studies have also shown that oxidative stress plays a key role in the development of AKI. For example, in a mouse model of renal I/R injury, heme oxygenase-1 knockout (HO-1^−/−^) mice were found to be more sensitive to I/R injury, while increasing the incidence of renal injury and mortality rate (Tracz et al., 2007). Oxidative stress further leads to downstream effects such as inflammatory damage, necrosis, and apoptosis. During oxidative stress, TMP mainly inhibits ROS generation and activates the antioxidant system. Liu *et al.* studied the protective effect of TMP on cisplatin-induced nephrotoxicity in rats, using ligustrazine for 7 consecutive days of intraperitoneal injection, starting from 2 days before a single intravenous injection of cisplatin. The results showed that cisplatin increased the levels of MDA, NOS, and NO, while the levels of GSH, GST, and SOD decreased. These changes were reversed by TMP treatment (Liu et al., 2008). Nrf2 is an important regulator of the antioxidant system that can neutralize the activation of cellular oxidative stress. Under basic conditions, the Keap1/Nrf2 complex is easily degraded by ubiquitination. However, under oxidative stress conditions, Keap1 is oxidized, and Nrf2 is introduced into the nucleus and binds to the antioxidant response element in the gene promoter region to initiate the transcription of a series of antioxidant factors (Saito, 2013; Suzuki and Yamamoto, 2015). Michel *et al.* found that TMP pretreatment significantly activated the Nrf2 defense pathway in rats with nephrotoxicity induced by cisplatin indicated by the increase in levels of Nrf2 and downstream antioxidant enzymes such as HO-1 and NQO1 in the kidney. This also shows that TMP inhibits cisplatin-induced oxidative stress by activating the Nrf2 defense mechanism (Michel and Menze, 2019). However, the regulation of Nrf2 and HO-1 signals by TMP is obviously complex. For example, as a response biomarker for arsenic exposure in various types of cells, HO-1 was observed downregulated by TMP pretreatment in arsenic-induced nephrotoxicity cell model, so did Nrf2 (Gong et al., 2016). We speculated that the reasons for the above contradictory results are multifaceted and complicated. The protective effect of Nrf2 in the kidney is affected by its activation degree and duration, and there might be a delicate balance. Studies have shown that in mice with renal tubule-specific knockout of Keap1, moderate activation of Nrf2 might reduce the damage caused by ischemia or nephrotoxic substances, while excessive and continuous activation of Nrf2 loses this protective effect (Noel et al., 2016; Tan et al., 2016; Nezu et al., 2017). Moreover, the transcription of the HO-1 gene is complicated and might not only be regulated by Nrf2. For example, sodium arsenite has been shown to cause BACH1-specific HO-1 induction independent of Nrf2 (Reichard et al., 2016). Additionally, there is a functional κB element in the promoter of mouse HO-1 gene, which might be the mechanism of HO-1 upregulation *in vivo* mediated by NF-κB subunits p50 and p65 (Li et al., 2009). Our previously data indicated clearly that arsenic-induced HO-1 expression is mediated by multiple pathways, and the corresponding transcription factors includes Nrf2, NF-κB AP-1, p38 MAPK, and JNK (but not ERK) (Gong et al., 2016). As an organ rich in mitochondria, kidney is very susceptible to oxidative stress mediated damage, thus reducing mitochondrial-derived ROS might be another important way to protect kidney against oxidative stress injury (Gorin, 2016). Our previous study also found that TMP could improve abnormal mitochondrial dynamics and regulate mitochondrial damage in contrast-induced nephropathy (CIN) (Gong et al., 2019). In addition, oxidative stress also interacts with a variety of pathological processes in the AKI process, including inflammation and apoptosis, which are discussed below. Therefore, TMP has the therapeutic potential of antagonizing oxidative stress in AKI caused by various etiologies.

### TMP Improves Inflammation

Inflammation is a physiological process that protects the body from acute damages such as ischemia, pathogens, or toxins. Inflammation is believed to play an important role in the pathogenesis of AKI. Basically, all immune cells, such as neutrophils, monocytes/macrophages, and NK cells are involved in the pathogenesis of AKI to varying degrees (Rabb et al., 2016). Activation of the inflammatory process in AKI is caused by multiple pathways. In models of ischemia, sepsis, and nephrotoxicity, the initial damage occurs in the tubular epithelium and vascular endothelial cells (Akcay et al., 2009). The above-mentioned damage induces the production of inflammatory mediators such as inflammatory factors, chemokines, and adhesion factors (TNF-α, TGF-β, IL-6, IL-1β, IL-18, CCL2, MCP-1, ICAM-1, and P-selectin), which help recruit leukocytes to the kidney. Neutrophils, macrophages, and lymphocytes infiltrate the injury site (McWilliam et al., 2021). In addition, oxidative stress can promote inflammation, and cell damage caused by inflammation further aggravates oxidative stress (Tucker et al., 2015). In the tetracycline-induced AKI rat model, the use of mitochondrial-targeted antioxidants significantly reduced the accumulation of dendritic cells and T cells in the kidney tissue, suggesting that mitochondrial-derived ROS are involved in antigen presentation and T-cell activation (Gentle et al., 2013). Under oxidative stress, NADPH oxidase (NOX) can interact with Toll-like receptor 4 (TLP4) to directly activate the nuclear transcription factor NF-κB pathway, leading to an increase in the transcription of downstream inflammatory mediators and further increasing inflammation (El-Benna et al., 2016). Most of the studies in Table 1 show an inhibitory effect on the level of inflammatory mediators, and the regulation of the NF-κB pathway is the key to TMP. NLRP3 is a member of the nucleotide-binding oligomerization domain-like receptor protein family (NLRPs) and is a common inflammasome. It promotes the maturation of the pro-inflammatory factors IL-1β and IL-18 by activating caspase-1 (Liston and Masters, 2017; Shi et al., 2017). The expression of signal sensing receptors such as TLRs and TNFRs and downstream gene expression proteins such as NF-κB and ROS is involved in the activation of NLRP3 (Xue et al., 2019). Many studies have shown that the NLRP3 inflammasome and its downstream apoptosis and inflammation play important roles in the occurrence and development of AKI (Bakker et al., 2014; Shen et al., 2016). Sun *et al.* explored the protective effect of TMP on renal ischemia-reperfusion injury in rats and its potential mechanism. The expression level of NLRP3 in NRK-52E cells increased after hypoxia and glucose deprivation, and decreased significantly after TMP treatment (Sun et al., 2020). As an important member of the CC subfamily of chemokines, CCL2 is also called monocyte chemotactic protein-1 (MCP-1). CCL2 is formed under pathological conditions such as pro-inflammatory stimuli (IL-8, TNF-α, and LPS stimulus). It usually binds to the extracellular specific ligand CCR2 to mediate the migration and activation of a variety of inflammatory cells (Kawaguchi-Niida et al., 2013). Our previous study found that the abundance of CCL2 and CCR2 in the renal tubules of rats with contrast-induced nephropathy (CIN) increased, accompanied by an increase in the concentration of IL-6 and TNF-α in the kidney and serum, and TMP could inhibit the CCL2/CCR2 pathway activation (Gong et al., 2019). The peroxisome proliferator-activated receptor (PPAR) is a member of the superfamily of nuclear transcription factors activated by ligands (Wu et al., 2018). PPAR-γ can inhibit the inflammatory response by competing with the inflammatory signaling pathway and the production of inflammatory mediators such as activator protein-1 (AP-1) and NF-κB (Ju et al., 2020). Studies have found that PPAR-γ expression is significantly reduced in cisplatin-induced acute kidney injury in rats, and TMP administration can significantly improve this change (Michel and Menze, 2019). In summary, TMP is a promising anti-inflammatory agent for treating AKI.

### TMP Inhibits Apoptosis

Apoptosis refers to the biochemical process of cell breakdown by a set of specific proteins that interact with each other and program death-inducing signals. Unlike necrosis, apoptosis does not cause inflammation (Kønig et al., 2019). When a cell receives an apoptosis signal, it activates the initial caspases through different signaling pathways, reactivates the effector caspases, and degrades related substrates, eventually leading to cell apoptosis (Chota et al., 2021). Since there are many apoptotic signaling pathways, the upstream regulation of caspases is also different. Bcl-2 family molecules are involved in upstream regulatory pathways for the reception and transmission of apoptosis signals. They mainly regulate apoptosis *via* the mitochondrial pathway. When pro-apoptotic proteins receive apoptosis signals, they can release cytochrome C (Cytc) from the mitochondria to activate downstream caspases, then causing apoptosis (Singh et al., 2019). The permeability of the mitochondrial membrane is regulated by Bcl-2 family proteins. In renal epithelial cells, Bcl-2 members Bax and Bak cause an increase in membrane permeability, while Bcl-2 and Bcl-XL antagonize this “membrane attack” effect (Youle and Strasser, 2008). Intrarenal stress and ischemia both increase the ratio of Bax/Bcl2, which is the main determinant of cell death (Chien et al., 2005; Liu and Baliga, 2005). In most AKI models, the adjustment effect of TMP on the ratio of Bax/Bcl2 has been confirmed in many studies. Juan *et al.* showed that gentamicin significantly induced apoptosis in NRK-52E cells in a dose-dependent manner. TMP pretreatment can inactivate the activities of caspase-3, caspase-8, and caspase-9 stimulated by gentamicin, inhibit the release of Cytc, and increase the expression of Bcl-XL (Juan et al., 2007). Although renal tubular cell apoptosis is often reported in various AKI models, the upstream signaling pathways leading to apoptosis may be different (Havasi and Borkan, 2011; Linkermann et al., 2013). Although there are different initiation mechanisms, most apoptotic pathways cluster on the mitochondria. The endogenous mitochondrial apoptotic pathway begins with oxidative stress. ROS and other stress products enter the mitochondria with the Bax/Bcl-2 protein complex, promote the increase in mitochondrial permeability with other pro-apoptotic genes, and then release Cytc (Galluzzi et al., 2018). Therefore, the anti-oxidative stress ability of TMP can regulate the mitochondrial apoptosis pathway from the source. There is also a close relationship between apoptosis and mitochondrial dynamics. Previous studies have shown that in the early stage of apoptosis, Bax is transferred from the cytoplasm to the mitochondria before the caspases are activated, and, at the same time, dynein-related protein 1 (DRP1) is also transferred from the cytoplasm to the mitochondrial division site and then mediates mitochondrial division (Suen et al., 2008). Inhibiting the activity of Drp1 not only inhibits mitochondrial division but also inhibits the activation and apoptosis of caspases (Hoppins and Nunnari, 2012). In addition, high expression of mitochondrial outer membrane fusion proteins Mfn1 and Mfn2 can also inhibit apoptosis (Jian et al., 2018). Our previous found that TMP could improve abnormal mitochondrial dynamics by upregulating Mfn2 and downregulating Drp1 and alleviating the apoptosis of tubule epithelial cells caused by contrast agents (Gong et al., 2019). In addition, the external pathway of apoptosis mediated by TNFR may also be involved in renal tubular cell apoptosis in ischemic and septic AKI (Cunningham et al., 2002; Linkermann et al., 2014). TNFR knockout mice are resistant to cisplatin-induced AKI, supporting this pathogenesis (Ramesh and Reeves, 2004). TMP can simultaneously regulate the upstream ligand (TNF-α) and downstream signaling pathways (JNK and NF-κB) of the TNFR-mediated apoptosis pathway.

### TMP Adjusts Autophagy

Autophagy is a process in which a double-membrane autophagosome encapsulates cytoplasm, organelles, and protein polymers and is transported to lysosomes for catabolism (Youle and Narendra, 2011). Under normal physiological conditions, low levels of basal autophagy maintain cell homeostasis by removing damaged proteins and organelles. The autophagy pathway is upregulated in stress states such as cell starvation, hypoxia, and endoplasmic reticulum stress (Feng et al., 2014). Autophagy is non-selective, but it can also selectively degrade damaged organelles such as mitophagy to clear damaged mitochondria. The formation of autophagosomes depends on the coordination of autophagy-related proteins, which mainly include the ULK1/2 complex, Beclin-1/class III PI3K complex, and autophagy-related genes (ATG). LC3-II is located in pre-autophagosomes and autophagosomes, and its level increases with the increase in autophagosome membranes. The Beclin-1/class III PI3K complex promotes the nucleation of autophagosomes on the phagocytic vesicle membrane. Both LC3-II and Beclin-1 are markers for autophagy detection (Dancourt and Melia, 2014). There are many reports on the link between AKI and autophagy, most of which indicate the protective effect of autophagy on AKI. Studies have found that the expression of LC3 in proximal tubule cells of ATG5-deficient mice after renal I/R injury is inhibited, suggesting that basic autophagy has a protective effect against renal injury caused by I/R injury (Kimura et al., 2011). In the CI-AKI rat and cell models established with iohexol, it was found that the expression of autophagy marker LC3-II in renal tubular epithelial cells increased, the mitochondrial damage of renal tubular cells increased after the use of autophagy inhibitors, and apoptosis increased (Ko et al., 2016). Although most studies have found that autophagy activated in renal tubular epithelial cells of various AKI plays a protective role, a few studies have suggested that autophagy aggravates cell damage in AKI. Chen *et al.* found that TMP could reduce renal I/R damage by enhancing autophagy, indicated by increased LC3-II/I ratio and Beclin-1 in kidney tissue (Chen et al., 2020). Another study found that TMP reduced inflammation in renal I/R injury and was related to the activation of autophagy (Jiang et al., 2020). Interestingly, in a study on CI-AKI, we found that the mechanism by which TMP protected the kidney from contrast agent damage was partly related to the inhibition of autophagy (Gong et al., 2019). The reason for this apparently contradictory result may be related to the different AKI models. Some studies have reported that autophagy induces cell metabolism imbalance and induces cell death in renal tubular epithelial cells induced by contrast agents, and this result can be attenuated by curcumin (Buyuklu et al., 2014). This shows that the role of autophagy in AKI is still controversial. As an upstream regulator of autophagy induction, ROS not only induces autophagy through the mitochondrial pathway but also induces mitophagy through the signaling pathway mediated by HIF-1 (Scherz-Shouval and Elazar, 2007; Zhang et al., 2008). The regulation of oxidative stress and HIF-1 by TMP is also one of the ways to regulate autophagy. In addition, there is an interaction between autophagy and apoptosis. In response to stress such as hypoxia, autophagy can prevent cells from triggering the apoptotic pathway by degrading misfolded proteins and damaged organelles. The inhibitory effect of Bcl-2 family proteins on autophagy in renal tubular cells has been confirmed in many experiments. In Bcl-2/GFP-LC3 transgenic mice, autophagy induced by ischemia-reperfusion was attenuated (Isaka et al., 2009). Studies have shown that enhancing the expression of Bcl-XL in the kidney is sufficient to inhibit autophagy induction and apoptosis (Chien et al., 2007). Regarding the mechanism by which Bcl-2 downregulates autophagy, it is generally believed that Bcl-2 family proteins bind Beclin-1 through the BH3 domain, blocking the necessary process of autophagosome formation. The details of the simultaneous regulation of autophagy and apoptosis by TMP are still unclear, and this may be a promising research direction. In short, the mechanism by which TMP interferes with autophagy in AKI is unclear, and there are still controversies.

## The Potential of TMP Prevents CKD and Renal Fibrosis

AKI and CKD are very tightly linked by each other. Many studies suggested that AKI is also an important inducement of chronic kidney disease (CKD) (Horne et al., 2017; See et al., 2019). There are many published data of TMP against CKD as well as renal fibrosis. In China, several TMP injections have been used to treat CKD in clinical, especially in diabetic nephropathy patients (Wang et al., 2012). Cao *et al.* reported that TMP had an inhibitory effect on the proliferation of human renal interstitial fibroblasts in a time- and concentration-dependent manner (Cao et al., 2006). Unilateral ureteral obstruction (UUO) model is a classic model for studying renal fibrosis, *Yuan et al.* reported that TMP treatment could reduce the score of interstitial collagen deposition, the density of macrophages, and the mRNA expressions of TGF-β1 and CTGF in this rat model (Yuan et al., 2012). The matrix accumulation caused by the reduction of the ratio of MMPS/TIMPS is the basic pathophysiological process of renal interstitial fibrosis (Kelly et al., 2010). Studies had found that TMP could inhibit the high expression of TIMP-1 and the imbalance of MMP-9/TIMP-1 ratio in UUO model rats, and thereby slow the progression of renal fibrosis (Li et al., 2017). TGF-β/Smad3 is the main pathway of renal fibrosis, and Smad7 could block the phosphorylation of Smad3, thereby limiting the effect of TGF-β (Meng et al., 2016; Chen et al., 2018). The results of Lu *et al.* showed that TMP could reduce the content of TGF-β1 in kidney tissue and restore the expression levels of Smad reverse regulators Smad7 and SnoN protein (Lu et al., 2009). In addition, aristolochic acid is very toxic to kidney, which would cause tubulointerstitial damage and renal fibrosis, and TMP has been reported to reduce the kidney damage caused by aristolochic acid in rats (Wang et al., 2006).

## Conclusion and Perspective

Considering the importance of oxidative stress and inflammation in AKI, the application of TMP in AKI treatment deserves attention. The present study mainly focuses on the experimental research of TMP in preventing AKI, and aims to synthesize the current knowledge in this field, concurrently, this study also briefly sums up the effects of TMP against renal fibrosis and CKD. Based on the collected data, TMP not only improves kidney function, reduces the level of kidney injury markers (including kim-1, CysC, UNAG, and UGGT), but also decreases the degree of pathological damage in kidney. Although the pathological mechanisms of AKI caused by various factors are different, the preventive effects of TMP against AKI are inseparable from the following four processes: oxidative stress, inflammatory mediators, apoptosis, and autophagy. These data support the potential application of TMP as a new therapeutic drug for AKI. It should be noted that these data mainly are preclinical studies, and the clinical application of TMP in AKI treatment still needs more rigorous clinical research data. As mentioned above, our group has been focusing on the basic experimental research of TMP against CI-AKI for more than 10 years (Gong et al., 2013, 2019; Gong, 2018; Norgren and Gong, 2018), and we hope these basic experimental research data should promote the followed clinical research progress of TMP treating CI-AKI and other types of AKI, not just for anti-renal fibrosis and the treatment of CKD.

## Glossary

ALP: alkaline phosphatase
ANP: acute necrotizing pancreatitis
ARS2: Arsenic response protein 2
BUN: blood urea nitrogen
CBS: cystathionine-beta-synthase
CIN: contrast-induced nephropathy
COX-2: cyclooxygenase-2
ET-1: endothelin-1
FoxO1: Fork-head box O1 transcriptional factor
GR: glutathione reductase
GSH: glutathione
GSSG: glutathione disulfide
GST: Glutathione-S-transferase
HAX-1: HS-1-associated protein
HMGB1: high mobility group box 1
HIF-1: hypoxia inducible factor-1α
HO-1: Heme oxygenase-1
ICAM-1: intercellular cell adhesion molecule-1
IL: interleukin
I/R: Ischemia-reperfusion
kim-1: kidney injury molecule-1
LDH: lactate dehydrogenase
LPO: lipid peroxidation
MAPK: Mitogen-activated protein kinase
MATs: methionine adenosyltransferases
MCP-1: monocyte chemoattractant protein
MDA: malondialdehyde
MICA: major histocompatability complex class I chain-related antigen A
NAG: N-acetyl-b-D-glucosaminidase
NF-κB: Nuclear factor-κB
NLRP3: nucleotide-oligomerization domain-like receptor 3
NMDARs: N-methyl-d-aspartate receptors
NO: Nitric oxide
NOD2: Nucleotide-binding oligomerization domain-containing 2
NOS: nitric oxide synthase
NQO1: NAD (P) H: quinone oxidoreductase 1
Nrf2: Nuclear factor erythroid derived-2
OGD: oxygen-glucose deprivation
PGI2: prostaglandin I2
PPAR-γ: peroxisome proliferator-activated receptor-gamma
ROS: reactive oxygen species
SAM: S-adenosylmethionine
SOD: superoxide dismutase
SrCr: serum creatinine
TBSA: total body surface area
TLR4: toll-like receptor 4
TNF-α: tumor necrosis factor
TOX: total antioxidant activity
TXA2: thromboxane A2
4-HNE: 4-hydroxynonenal
